# Addressing biodiversity loss by building a shared future

**DOI:** 10.1371/journal.pbio.3001690

**Published:** 2022-05-31

**Authors:** Roland G. Roberts, on behalf of PLOS Biology staff editors

**Affiliations:** Public Library of Science, San Francisco, California, United States of America

## Abstract

As the UN International Day for Biological Diversity enters its twentieth year, we take stock of recent developments and trends in biodiversity research and renew the call to build a better shared future for all life.

The 22^nd^ of May 2022 marked the twentieth anniversary of the United Nation’s International Day for Biological Diversity, which was instigated as an annual event in 2002, ten years after the adoption of the Convention on Biological Diversity. We therefore stand thirty years on from the formal recognition by the global community that the diversity of life on Earth is an asset that should be valued in its own right, and that humanity should endeavour to protect it.

*PLOS Biology* has been around for nearly twenty of those thirty years, publishing work that directly addresses questions of global biodiversity—how it arose, how it has changed in the past, how it is being affected by ongoing anthropogenic activity, and what we can do to protect it.

Strikingly, one of our most read and cited papers ever [1] was one that simply sought to establish how many species exist on the planet. The debate around this topic is ongoing; estimates of the total number of species vary by at least four orders of magnitude, and it has recently been suggested that massive diversity may lie underappreciated in the bacteria that live in and on the bodies of animals [2]. However, a paucity of data for many taxonomic groups hinders such census efforts. Considering that, even for some well-studied taxonomic groups, the current rates of extinction are unknown or seem to have been substantially underestimated [3, 4], global biodiversity is in jeopardy.

A cursory survey of biodiversity-related papers that have been published in the journal in the past year reveals several principal topics. In this issue alone, we feature work addressing the biodiversity of marine communities half a billion years apart [5, 6] and the use of artificial intelligence to automate the surveillance of threatened species [3].

Across the past year, one topic to emerge has been how biodiversity has changed in the past, with papers attempting to infer what forces, both biotic and abiotic, have driven these changes, often with the implicit or explicit expectation that we can learn lessons about future change. These papers include (in roughly temporal order) studies of ancient Ediacaran animal communities [5], end-Cretaceous sharks [7], Cenozoic snakes [8], and cold-water corals over the past 20,000 years [6] (Fig 1). A further paper leaves empiricism behind and presents a tool for using simulation to probe the drivers of biodiversity [9].

**Fig 1:**
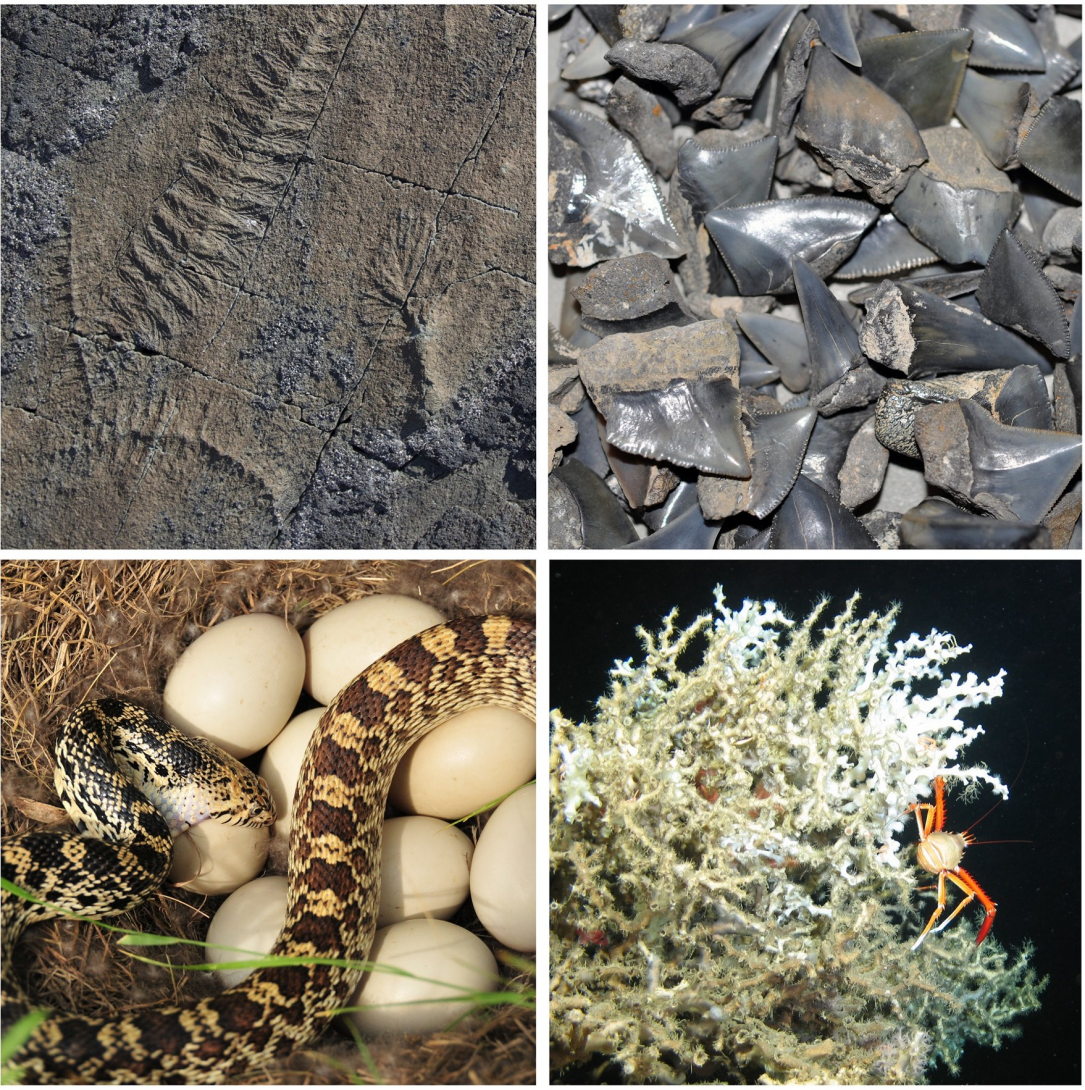
More than half a billion years of biodiversity. Top left: Fossils of Ediacaran creatures *Fractofusus* and *Plumeropriscum* from Newfoundland, Canada, subjects of a metacommunity analysis of shifts in biodiversity more than 540 million years ago [5]. Top right: Late Cretaceous sharks’ teeth from southern Sweden, which contributed to a new picture of how shark biodiversity was—and was not—impacted by the end-Cretaceous extinction event [7]. Bottom left: A bullsnake (*Pituophis catenifer sayi*) eating mallard eggs, illustrating a study that highlights the role of dietary complexity in snake biodiversity during the Cenozoic [8]. Bottom right: The cold-water coral *Lophelia pertusa* (with a squat lobster), one of several species of such corals whose fate over the last 20,000 years suggests sensitivity to food supply and oxygen levels [6]. Image credits: Charlotte Kenchington, Benjamin Kear, Tom Koerner/USFWS via Flickr, MARUM ROV Cherokee, respectively.

Another main topic to emerge is how best to assess current biodiversity and to target conservation effort. One study in this area reveals global and national inequities in monitoring species distributions [10], whereas a study of data in Australia—a nation with clear vested interest in its biological resources—shows that even there, many endangered species remain taxonomically undocumented, compromising their conservation [11]. Another paper proposes the use of machine learning to avoid the time-consuming and potentially biased reliance on human experts for judging extinction risk [3]. A fourth study intriguingly reveals the dangers of the dominant place that the English language holds in academic discourse, quantifying the loss of biodiversity knowledge incurred by an exclusive dependence on English language sources [12].

Finally, given a firmer grasp of the richness of life on earth and how this arose, we have the topic of solutions, ranging from the macroscopic strategies of optimising large areas of habitat protection [13] to the sub-microscopic—a proposal that molecular biology harbours “a vast potential for tackling climate change and biodiversity loss” [14].

The theme for this year’s UN International Day for Biological Diversity is “Building a shared future for all life.” In explaining this slogan, the organisation argues that “biodiversity is the foundation upon which we can build back better.” *Homo sapiens* arose as a product of the very processes mentioned above, one twig on the colossal and entangled tree of biodiversity. As humans, we simultaneously bear both the responsibility for a disproportionate destructive impact on the rest of the planet, and the knowledge and technology with which to mitigate it. We should now build back. Better.
